# Treatment of Nervous and Rheumatic Affections by Static Electricity

**Published:** 1874-06

**Authors:** 


					﻿Treatment of Nervous and Rheumatic Affections by Static Electricity. By-
Dr. A. Arthius. Translated from the French by J. H. Etheridge, M.D., Prof,
of General Therapeutics, Rush Medical College, Chicago. W. B. Keen, Cooke
& Co. 1874.
The author claims very great advantage in the use of static elec-
tricity, in its application for the cure of nervous, rheumatic and
other affections, over the general method now employed. The
application is new, and, we doubt not, superior to the usual man-
ner of administering electricity. It is, at least, worthy the atten-
tion of the profession, by whom, we trust, it will be thoroughly
tested.
Atlantic Monthly.—The July number of the “Atlantic”
comes to hand replete with its usual feast of “good things.” The
Atlantic is always “tip-top,” rich in contents and unexcelled
in its get-up. The “Rebel” George Cary Eggleston gives its read-
ers a few scenes behind “Confederate” times, which is good reading
for summer. Price, $4. H. O. Houghton & Co., 219 Washing-
ton street, Boston.
Retirement.—We learn from Dr. B. F. Dawson that he has
retired from the editorial management of the American Journal of
Obstetrics and Diseases of Women and Children, of which he was the
founder. Dr. Dawson is a fine scholar and accomplished medical
gentleman. The editorial brotherhood, no less than the profession,
will miss his valuable services. The Journal is now, we learn,
published by Wm. Wood & Co., N. Y.—but we never see it.
The Popular Science Monthly is the best and handsomest
science monthly in America—yea, in the world. It stands unri-
valed—a star among all similar publications. It deals with sub-
jects purely scientific, and is a sine qua non to the scientific reader
of America. It is published by that always reliable publishing
house, D. Appleton & Co., 549 and 551 Broadway, New York, at
§5 per annum.
Demorest’s Monthly is a ladies’ book of choice reading, and
filled with fashion plates beautiful and attractive to all.
				

